# Network Pharmacology-Based Strategy for the Investigation of the Anti-Obesity Effects of an Ethanolic Extract of *Zanthoxylum bungeanum* Maxim

**DOI:** 10.3389/fphar.2020.572387

**Published:** 2020-11-13

**Authors:** Ying Wang, Song Hong Yang, Keying Zhong, Ting Jiang, Mi Zhang, Hiu Yee Kwan, Tao Su

**Affiliations:** ^1^ International Institute for Translational Chinese Medicine, Guangzhou University of Chinese Medicine, Guangzhou, China; ^2^ School of Pharmacy, Jiangxi University of Traditional Chinese Medicine, Nanchang, China; ^3^ School of Chinese Medicine, Hong Kong Baptist University, Hong Kong, China

**Keywords:** *Zanthoxylum bungeanum* Maxim, network pharmacology, high-fat diet, obesity, apoptosis

## Abstract

Network pharmacology is considered as the next paradigm in drug discovery. In an era when obesity has become global epidemic, network pharmacology becomes an ideal tool to discover novel herbal-based therapeutics with effective anti-obesity effects. *Zanthoxylum bungeanum* Maxim (ZBM) is a medicinal herb. The mature pericarp of ZBM is used for disease treatments and as spice for cooking. Here, we used the network pharmacology approach to investigate whether ZBM possesses anti-obesity effects and reveal the underlying mechanism of action. We first built up drug–ingredient–gene symbol–disease network and protein–protein interaction network of the ZBM-related obesity targets, followed by Gene Ontology and Kyoto Encyclopedia of Genes and Genomes pathway enrichment analyses. The results highlight apoptosis as a promising signaling pathway that mediates the anti-obesity effects of ZBM. Molecular docking also reveals quercetin, a compound in ZBM has the highest degree of connections in the compound-target network and has direct bindings with the apoptotic markers. Furthermore, the apoptotic effects of ZBM are further validated in 3T3-L1 adipocytes and in the high-fat diet–induced obesity mouse model. These findings not only suggest ZBM can be developed as potential anti-obesity therapeutics but also demonstrate the application of network pharmacology for the discovery of herbal-based therapeutics for disease treatments.

## Introduction

Obesity has reached epidemic proportions globally. Based on the data reported by the Centers for Disease Control and Prevention, the prevalence of obesity in the United States has increased from 30.5 to 42.4%, and the prevalence of severe obesity has increased from 4.7 to 9.2% in the last decade (Agha and Agha, 2017). Asian countries such as China have 46% of adults being obese or overweight (Wang et al., 2019b). Moreover, the prevalence of obesity or overweight in youngsters and childhood is also increasing worldwide. The WHO reports that the number of obese children and adolescents had a tenfold increase, which had already reached 124 million in 2016, and 216 million children in the world were overweight.

Obesity is associated with many comorbid conditions and is the main risk factor for many noncommunicable diseases. Overweight and obesity are closely associated with polycystic ovary syndrome, which is an endocrine condition that causes enlarged ovaries, prevents proper ovulation, and reduces fertility (Barthelmess and Naz, 2014). Obesity is also the risk factor for type 2 diabetes, high blood pressure, heart disease and strokes, sleep apnea, osteoarthritis, fatty liver disease, kidney disease, and certain types of cancers such breast cancer, colorectal cancer, and kidney cancer (Pi-Sunyer, 2009). Every year, at least 2.8 million people are dying because of being overweight or obese. Obesity and its associated conditions cast a heavy burden on the health sector.

Recently, network pharmacology has been used to explore the therapeutic effects and therapeutic targets of Chinese medicinal herbs and bioactive compounds. The “network target, multi-components” concept of network pharmacology is the most suitable tool to explore the therapeutic effects of herbal medicine at the molecular level (Li and Zhang, 2013; Zhang et al., 2013). The network pharmacology approach is a new research paradigm that facilitates the development of evidence-based medicine and novel herbal-based drug discovery.

*Zanthoxylum bungeanum* Maxim (ZBM) is a Chinese medicinal herb; it belongs to the family Rutaceae and is widely distributed in south central and southwest China. ZBM has been recorded in “Shen Nong’s Herbal Classic” and “Compendium of Materia Medica” and is described as “hot, nontoxic.” In Chinese medicine practice, the mature pericarp of ZBM is used to treat colds, stomach and abdomen pains, vomiting, and diarrhea, while the seeds are used to treat edema, tumescence, and dyspnea due to phlegm and retained fluid. In Chinese Pharmacopoeia, ZBM is described as a medicinal herb for somebody who lost appetite. In our daily life, the mature pericarp of ZBM is also used in cooking as spice. It is both medicine and food. Pharmacological studies have demonstrated that ZBM has anti-obesity properties (Gwon et al., 2012), and some constituents occurring in ZBM, for example, quercetin (Kim et al., 2015), rutin (Yuan et al., 2017), and sanshool (Wang et al., 2019a) have been reported to exert anti-obesity effects. However, the anti-obesity mode and mechanism of action of ZBM are not fully understood.

**FIGURE 1:**
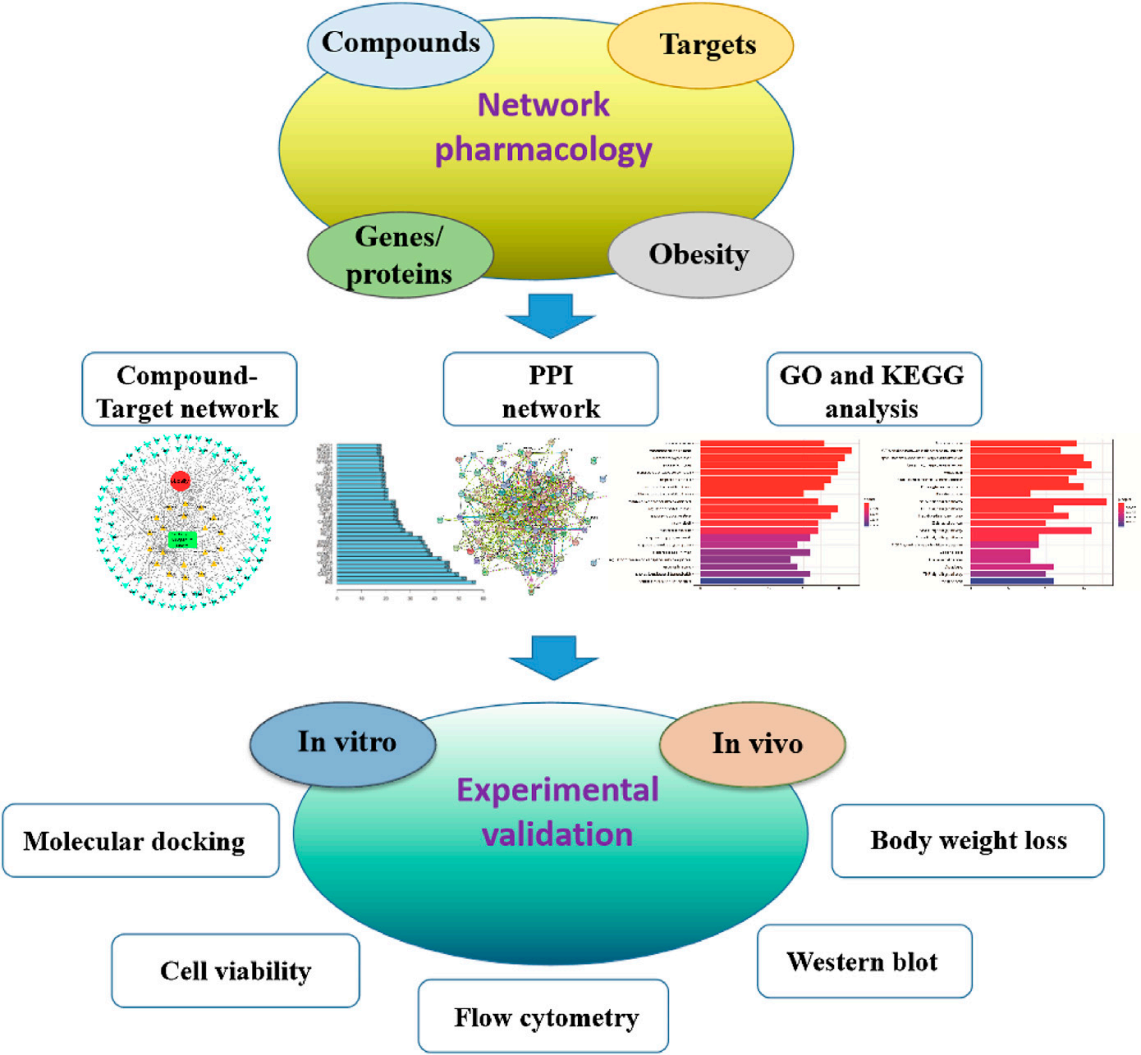
Flowchart showing the network pharmacological and experimental studies for the investigation of the anti-obesity effect of *Zanthoxylum bungeanum* Maxim.

In this study, we aimed to employ network pharmacology to explore whether the mature pericarp of ZBM possess anti-obesity effects and delineate the underlying mechanism of action. Furthermore, both *in vitro* and *in vivo* studies have been done to validate its anti-obesity effects. A schematic diagram is shown in Figure 1.

## Materials and Methods

### Screening of Bioactive Ingredients from the Mature Pericarp of ZBM

All of the ingredients containing in ZBM were obtained from Traditional Chinese Medicine Systems Pharmacology Database and Analysis Platform (TCMSP, http://tcmspw.com/tcmsp.php) (Ru et al., 2014), Chinese Academy of Sciences Chemistry Database (www.organchem.csdb.cn), TCM Database@taiwan (http://tcm.cmu.edu.tw), and Traditional Chinese Medicine Integrated Database (https://omictools.com/tcmid-too) (Yang et al., 2019). The effective components from ZBM are mainly filtered according to their oral bioavailability (OB) and drug-likeness (DL) indices. Absorption, distribution, metabolism, excretion, and toxicity modeling as a tool for rational drug design has significant effects in new drug discovery (Wang et al., 2015). OB is one of the most important pharmacokinetic parameters in the absorption, distribution, metabolism, excretion and toxicity characteristics of drugs, indicating the ratio of the oral drug to the oral dosage of the blood circulatory system (Xu et al., 2012; Liu et al., 2013). High OB values is often an important consideration for the development of bioactive molecules as therapeutic agents (Alam et al., 2015). In order to filter out compounds which are not likely to be drugs, the OB was calculated using in-house software OBioavail 1.1 (Liu et al., 2018). This software is based on a dataset of 805 structurally diverse drug and drug-like molecules that have been critically evaluated for their OB (%F) in humans. DL evaluation is used in drug design to evaluate whether a compound is chemically suitable for use as a drug and how a drug-like molecule is with respect to parameters that affect its pharmacodynamic and pharmacokinetic profiles, which ultimately impact its ADME properties (Walters and Murcko, 2002). The Tanimoto coefficient was used to evaluate the DL index of the molecules in ZBM using the following formula:

$$T\left(\alpha ,\beta \right)=\frac{\alpha \times \beta }{\alpha^{2}+\beta^{2}-\alpha \times \beta },$$

where *a* is the molecular property of the ZBM ingredient on the basis of Dragon software (www.talete.mi.it/products/dragon_description.htm) and *β* denotes the average molecular property for all drugs in the DrugBank database (www.drugbank.ca/) (Mauri et al., 2006). Hence, we further selected the major ingredients based on the literature to identify the potential therapeutic effects. Although some ingredients, such as volatile oil have lower DL values, they were selected because the effect had been experimental verification (Zheng et al., 2020). Meanwhile, the chemical information of these ingredients (structure, specification name, and CID number) for computational analysis was also collected according to the PubChem (https://pubchem.ncbi.nlm.nih.gov/) and DrugBank (https://www.drugbank.ca/drugs).

### Identification of ZBM-Associated Molecular Targets

The potential molecular targets of ZBM were predicted using the TCMSP (Ru et al., 2014), SwissTargetPrediction (Gfeller et al., 2014), and the Search Tool for Interacting Chemicals (Szklarczyk et al., 2016).

### Identification of Obesity-Associated Molecular Targets

The obesity-associated targets were comprehensively collected from four databases including Therapeutic Target Database, Kyoto Encyclopedia of Genes and Genomes (KEGG), the Comparative Toxicogenomics Database, and GeneCards v4.9.0 (www.genecards.org/).

### Ingredient–Target Network Construction

The obtained drug-related targets and the disease-related targets were intersected, and a Venn diagram of the intersected gene symbols was obtained. Then, a complex information network was constructed based on the interaction of drug (ZBM), components, gene symbol, and disease (obesity). Cytoscape 3.7.1 software (Shannon et al., 2003) was used for visual analyses of the drug–component–target–disease network.

### Protein–Protein Interaction Network Construction

STRING online database (https://string-db.org/) (Hsia et al., 2015) was applied to obtain the PPI data of the molecular targets of ZBM, where the parameter organism was set to *Homo sapiens*, and other basic settings were the default value. Cytoscape software was employed to establish the PPI relationship network and perform topological analysis.

### Enrichment of Gene Ontology and Kyoto Encyclopedia of Genes and Genomes Pathways

The GO analysis and KEGG pathway enrichment were employed by using Bioconductor (R) v3.8 bioinformatics software (http://bioconductor.org/). Terms with expression analysis systematic explorer scores of ≤0.05 were collected for functional annotation clustering. The pathway enrichment analysis was performed using the KEGG database to verify the functional categories of statistically significant genes (*p* < 0.05). Terms with thresholds of count of ≥2 and Expression Analysis Systemic Explorer scores of ≤ 0.05 were screened for functional annotation clustering.

### Network Construction and Analysis

The compound–target network was generated by linking bioactive constituents and putative targets. Based on the predicted targets and the predicted obesity-related signaling pathways, a target–pathway network was established. The compound–pathway network was constructed based on all the compounds and the signaling pathways. In our network, the nodes represent the candidate compounds, potential targets, or signaling pathways, while the edges represent the compound–target or target–pathway interactions. Cytoscape software was used to construct the network.

### Computational Validation of Ingredient–Target Interactions

To further evaluate the results obtained in systemic pharmacologic analyses, quercetin, a compound in ZBM with the highest degree of connection among all the obesity-related targets, was selected for the test of its apoptotic effects in adipocytes. The three-dimensional (3D) structures of caspase 3 (PDB ID: 2XYH), Bcl-2 (PDB ID: 2W3L), and Bax (PDB ID: 5W63) were downloaded from RCSB Protein Data Bank (http://www.rcsb.org/pdb). In addition, we also performed the docking assay with three inhibitors of caspase 3, Bax, and Bcl-2 as positive controls to each target to verify whether our model is robust and reliable. The 3D structure of quercetin was drawn by ChemBioDraw Ultra 14.0 and ChemBio3D Ultra 14.0 software. Structures of the compounds were sketched using MarvinSketch (www.chemaxon.com), and ligand molecules were converted from 2D to 3D using ChemBioDraw Ultra 14.0 and ChemBio3D Ultra 14.0 software. The docking study was performed using AutoDock Vina (Trott and Olson, 2011), and input files necessary for AutoDock program were prepared using AutoDockTools (Morris et al., 2009). The size of the grid box in AutoDock Vina was kept as 40 × 40 × 40 for X, Y, and Z, and the default setting was kept for energy range. The automated program yielded nine possible conformations with distinguished binding energy for each ligand output. The final model was selected based on the binding affinity and molecular contacts. The molecular contacts were calculated using the program CONTACT available in CCP4 suite (Winn et al., 2011). Docked complexes were analyzed, and figures were rendered using PyMOL (www.pymol.org).

### Cell Culture and Reagents

3-(4,5-dimethylthiazol-2-yl)-2,5-diphenyltetrazolium bromide (MTT) and DMSO were purchased from Sigma Chemicals Ltd. (St. Louis, MO, USA). Antibodies against cleaved PARP (No. 5625 s), cleaved caspase 3 (No. 9661 s), and cleaved caspase 7 (No. 8438 s) were purchased from Cell Signaling Technology (Beverly, MA, USA); cleaved caspase 6 (No. ab108335), cleaved caspase 8 (No. ab108333), cleaved caspase 12 (No. ab62484), Bax (No. ab182733), and Bcl-2 (No. ab182858) were purchased from Abcam (Cambridge, MA, USA); Mcl-1 (No. sc-819) and *β*-actin (No. sc81178) were obtained from Santa Cruz Biotechnology (Santa Cruz, CA, USA). Their corresponding secondary antibodies and protein marker were supplied by Bio-Rad (Hercules, CA, USA). All materials for cell culture were obtained from Life Technologies Inc (GIBCO, USA).

### Extraction of Mature Pericarp of ZBM

ZBM, originated from Sichuan province, China, was purchased from the Chinese Medicine Clinic of the Hong Kong Baptist University and authenticated in accordance with the corresponding monograph in the Chinese Pharmacopoeia by the corresponding author. Voucher specimen of ZBM (No. 170901) was deposited at the School of Chinese Medicine, Hong Kong Baptist University. The mature pericarp of ZBM (100 g) was reflux-extracted twice with 50% ethanol (1:10, w/v) for 2 h each. The combined extracts were filtered after cooling and then concentrated under reduced pressure to remove ethanol. The powdered extracts (yield: 11.87%, we name the extract ZBM hereafter) were obtained by lyophilizing the concentrated samples with a Virtis Freeze Dryer (The Virtis Company, New York, USA).

### UPLC/Q-TOF-MS Analysis

Liquid chromatography was performed on an Agilent 1200 system coupled with an ACQUITY UPLC T3 C18 column (2.1 mm × 50 mm I.D., 1.8 μm) maintained at 32°C. Elution was performed with a mobile phase of A (0.1% FA in water) and B (0.1% FA in ACN) under a gradient elution of 10–20% B at 0–5 min, 20–50% B at 5–10 min, 50–70% B at 10–25 min, and 70–100% B at 25–32 min was employed. The flow rate was 0.4 mL/min, and the injection volume was 5 μL. Mass spectrometric detection was carried out on an Agilent 6540 Q-TOF mass spectrometer (Hewlett Packard, Agilent, USA) with an electrospray ionization interface. The positive ion mode was used with the mass range setting at m/z 100–1,700. Optimized ionization conditions were as follows: gas temperature, 300°C; drying gas (N_2_) flow rate, 8 L/min; nebulizer, 40 psi; sheath gas temperature, 350°C; sheath gas flow, 8 L/min; capillary voltage, 4,500 V; fragmentor, 175 V; skimmer voltage, 65 V; and Octopole RF peak, 600 V. Data were collected with LC-MS-QTOF MassHunter data acquisition software ver. A.01.00 (Agilent Technologies) and analyzed with Agilent MassHunter qualitative analysis software B.06.00, respectively. The peaks were tentatively identified by matching with empirical molecular formulae and mass fragments.

### 3T3-L1 Preadipocyte Differentiation

3T3-L1 preadipocytes (ATCC) were induced to differentiate into mature white adipocytes with differentiation-inducing medium containing 1 mM dexamethasone, 0.5 mM isobutylmethylxanthine, and 1.67 mM insulin in Dulbecco’s modified Eagle’s medium with 10% FBS for 4 days before switching to Dulbecco’s modified Eagle’s medium with only 10% FBS and 10 μg/mL insulin for an additional 3 days (Su et al., 2020).

### Cell Viability Assay

Cell viability was determined by the MTT assay. Briefly, 3T3-L1 cells were seeded into a 96-well plate and treated with ZBM at the indicated concentrations for 24 or 48 h. Vehicle served as control. After the treatment, MTT solution (5 mg/mL) was added and incubated with the cells for another 4 h at 37°C in dark. Dimethylsulfoxide (Sigma-Aldrich) was then used to dissolve the formazan precipitate. The absorbance of each well was measured at wavelength 570 nm in a microplate reader (Bio-Rad Laboratories). Cell viability was calculated according to the absorbance of each well with the following formula: cell viability (%) = [(A570 sample−A570 blank)/(A570 control−A570 blank)] ×100%, where A570 sample, A570 blank, and A570 control stand for the absorbance of treatment group, blank group (no cells), and control group (vehicle), respectively.

### Western Blot Assay

After treatments, 3T3-L1 cells, subcutaneous adipose tissues, or visceral adipose tissues were collected and lysed for 30 min on ice with lysis buffer. Samples were centrifuged at 15,000 rpm for 10 min at 4°C, and total protein concentrations were measured by using a Pierce BCA Protein Assay Kit (Thermo Fisher Scientific) and then denatured. Aliquots of 20–40 µg of cell or tissue lysates were separated in a 6–12% sodium dodecyl sulfate polyacrylamide gel along with PageRuler^™^ Prestained Protein Ladder (Thermo Scientific) and transferred onto a polyvinylidene difluoride membrane preactivated by methanol. The membrane was blocked for 2 h at room temperature with 5% nonfat milk powder dissolved in TBST (0.1% Tween-20 in TBS), and then incubated with primary antibodies against cleaved PARP, cleaved caspase 3, 6, 7, 8, and 12, Bax; Bcl-2; Mcl-1; or GAPDH for 12 h in 4°C. The secondary antibodies were diluted in TBST containing 5% milk and incubated for 1 h at room temperature. The immune-reactive targets were detected by an ECL Western Blotting Substrate Kit (Thermo Fisher Scientific). Band density was analyzed by ImageJ software and normalized with internal control.

### Quantification of Apoptosis

Apoptotic cells were assessed using annexin V-fluorescein isothiocyanate apoptosis detection kit I (BD, Bioscience) following the manufacturer’s instruction. Samples of 100,000 stained cells were analyzed using flow cytometry (Leica TCS SP8).

### Animal Handling

All the animal studies were approved and performed according to the guidelines of the Department of Health HKSAR, and animal research ethics panel in the Hong Kong Baptist University. C57 male mice of 4–5 weeks old were purchased from the Chinese University of Hong Kong. Mice were randomly selected to have either control diet (D12450J Research Diets) or high-fat diet (D12762 Research Diets) which was used to induce obesity. Both diet and water were supplied *ad libitum*. Body weight of each mouse was recorded every week. After 10 week of dietary intervention, the body weight of high-fat diet (HFD)–fed mice and comparable control diet (CD)–fed mice were significantly different, indicating the diet-induced obesity (DIO) mouse models were established. The DIO mice were then given either ZBM (4 g/kg) or vehicle control by oral administration. Behavioral changes, body weight, and food intake of these mice were recorded every day.

## Results

### Identification of Bioactive Components From ZBM

According to the UPLC/Q-TOF-MS analyses, 13 bioactive compounds in ZBM were tentatively identified by matching with the empirical molecular formulae and mass fragments (Supplementary Figure S1). Details were shown in Supplementary Table S1. In addition, according to the high-performance liquid chromatography analysis, two characteristic constituents in ZBM, such as hydroxy-*α*-sashool and hydroxy-*β*-sanshool, were also identified with reference standards (Supplementary Figure S2). After combining with the identified compounds and the compounds collected in three databases, a total of eighty-four candidate compounds were identified (Supplementary Table S2). To identify the active ingredients of ZBM, two classical ADME parameters, OB and DL, were used for screening. OB ≥15% and DL ≥0.1 were considered to have relatively better pharmacological properties. Although some ingredients do not meet the screening criteria, they have clinical therapeutic effects; we also kept these ingredients in our research for a comprehensive analysis. For example, rutin had a low OB, while it is a major and active constituent of ZBM (Zhang et al., 2017). Increasing studies have demonstrated that rutin reduces obesity by activating brown fat (Yuan et al., 2017). Besides, in HFD-induced obese rats, rutin increases muscle mitochondrial biogenesis coupled with AMP-activated protein kinase activation and reduces body weight by increasing brown adipose tissue mitochondrial biogenesis (Seo et al., 2015). Another compound, hyperoside, has a low DL, but it has a beneficial effect on the controlling body weight because it inhibits adipogenesis (Berkoz, 2019). It is important to note that although the pharmacokinetic parameters of these components are relatively low, they are bioactive and therefore are considered as the candidate ingredients. Hence, we expanded the screening standard procedure beyond the ADME principles; we assumed that if the candidate components in ZBM intersected with the obesity targets, they were considered as the active components. Therefore, as shown in Table 1, a total of 20 ingredients were selected as active ingredients in ZBM.

**TABLE 1:** A total of twenty active ingredients were obtained from ZBM.

No	CAS	Name	References
1	7149-26-0	Linalyl anthranilate	(Dharmawan et al., 2008)
2	469-92-1	CLOVENE	(Cardeal et al., 2006)
3	577-27-5	Ledol	(Dhami et al., 2019)
4	73464-47-8	β-Gurjunene	(Cardeal et al., 2006)
5	13744-15-5	β-Cubebene	(Sekiwa-Iijima et al., 2002)
6	83-95-4	Skimmianin	(Abe et al., 1973)
7	520-34-3	Diosmetin	(Cardeal et al., 2006)
8	6750-60-3	Spathulenol	(Shafi et al., 2000)
9	83-46-5	β-sitosterol	(Tsai et al., 2000)
10	482-36-0	Hyperoside	(Ahsan et al., 2000)
11	531-59-9	Herniarin	(Sekiwa-Iijima et al., 2002)
12	581-31-7	Suberosin	(Ahsan et al., 2000)
13	17156-84-2	Oleic acid	(Li et al., 2001)
14	117-39-5	Quercetin	(Ahsan et al., 2000)
15	153-18-4	Rutin	(Cho et al., 2003)
16	83883-10-7	Hydroxy-α-sashool	(Rong et al., 2016; Li et al., 2019)
17	97465-69-5	Hydroxy-β-sanshool	(Rong et al., 2016; Li et al., 2019)
18	78886-66-5	Hydroxy-γ-sanshool	(Rong et al., 2016; Li et al., 2019)
19	138-86-3	Limonene	(Sun et al., 2020)
20	78-70-6	Linalool	(Sun et al., 2020)

### Identification of the Related Targets and Gene Symbols of the Ingredients in ZBM

All the targets of the ingredients in ZBM were obtained from the TCMSP (http://lsp.nwu.edu.cn/tcmsp.php). After removing the redundant information, only those targets that can interact directly with each component in the ZBM are retained. Then, the targets were transformed using the UniProt knowledge database (www.uniprot.org), and the duplicated gene symbols were deleted. A total of 20 ingredients in ZBM and 101 known molecular targets of these ingredients were highlighted (Supplementary Table S3).

### Acquisition of Known Therapeutic Targets for Obesity

A total of reported 7,268 obesity-related therapeutic targets were collected from the GeneCards database. Besides, 11 reported therapeutic targets for obesity were obtained from the OMIM database. After removing duplicated gene symbols, a total of 7,187 therapeutic targets for obesity were shortlisted (Supplementary Table S4).

### Analyses of the Drug–Ingredient–Gene Symbol–Disease Network

**FIGURE 2:**
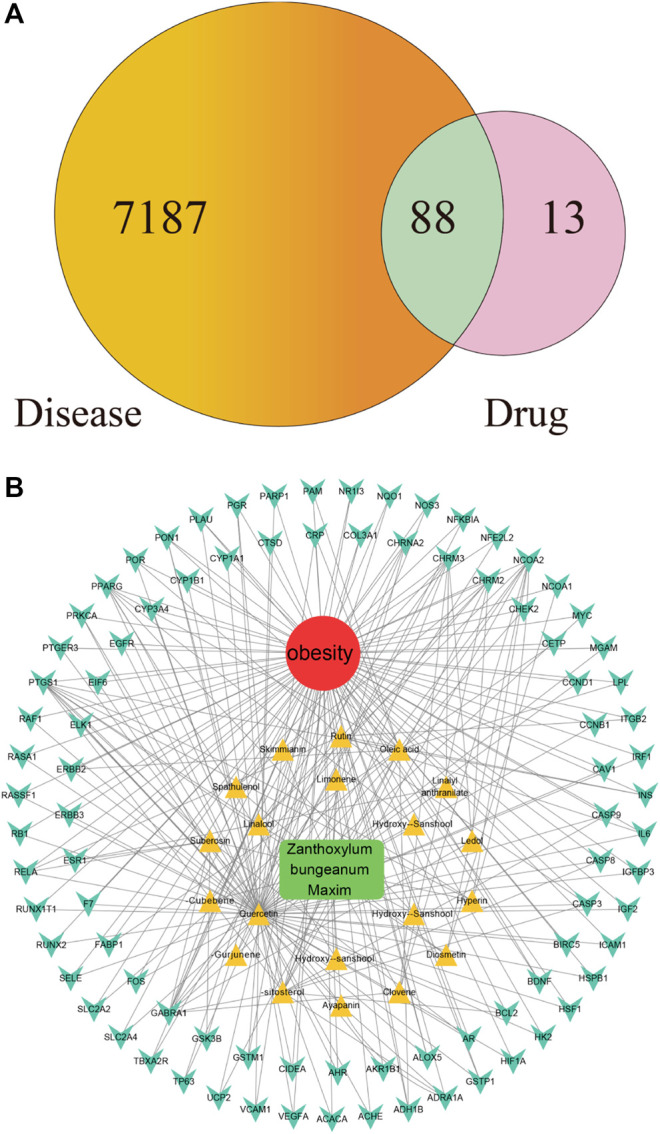
**(A)** Overlapping gene symbols between the disease and *Zanthoxylum bungeanum* Maxim (ZBM) **(B)** D-I-G-D network. The green node represents ZBM, and the red node represents obesity, twenty yellow nodes represent the active ingredients in ZBM, and the 88 cyan nodes represent the overlapping gene symbols between the disease and ZBM. The edges indicate that nodes can interact with each other.

As shown in Figure 2A, among the 7,275 gene symbols related to the disease and the 101 gene symbols related to the drug, 88 of them are overlapped, suggesting that these 88 genes may be the molecular targets that mediate the anti-obesity effects of ZBM. Details of the 88 gene symbols were shown in Supplementary Table S5.

Chinese herbals present a range of pharmacological activities by acting on a wide range of molecular targets. We next investigated the potential mechanisms of action underlying the anti-obesity effects of ZBM. Based on the compounds and highlighted molecular targets, we constructed a D-I-G-D network using Cytoscape. As shown in Figure 2B, green node represents ZBM, red node represents obesity, 20 yellow nodes represent the active ingredients of ZBM, and 88 cyan nodes represent the overlapping gene symbols between obesity and ZBM. The edges indicate that the nodes can interact with each other. Further network analyses were performed by evaluating the centralization and heterogeneity. The centralization and heterogeneity of the network were 0.778 and 2.260, respectively. The network indicated the potential relationship between the compounds and the targets, which implied the potential pharmacological mechanisms of ZBM or the compounds in reducing obesity. The nodes with the highest degree of connection to other compounds or targets represent hubs in the entire network, which are the potential drugs or targets. For example, the compound with the highest degree of connections was quercetin (degree = 71). Rutin and herniarin (ayapanin) also have high degree of connections of 10 and 5, respectively. These findings suggest that a single compound affects multiple targets, and some of the active ingredients in ZBM could exert anti-obesity effects via multiple targets. Quercetin is an active compound of ZBM; it has anti-obesity effects, and it induces HO-1 expression to increase hepatic mitochondrial oxidative metabolism (Kim et al., 2015). Furthermore, quercetin reduces the expression of the key adipogenic factor C/EBP *α* and inhibits lipogenesis by downregulating the expression levels of fatty acid synthase and acetyl-CoA carboxylase in HFD-induced obesity rats (Moon et al., 2013). Quercetin also upregulates uncoupling protein-1 expression that transiently increases energy expenditures in the HFD-fed mouse model (Stewart et al., 2008). Regarding the target analysis, AKT1, IGF1R, and FGFR1 were separately linked to 62 compounds; MTOR, BRAF, and MAP2K1 were separately connected to 61 compounds. These findings indicated that different compounds could target a single gene in a collaborative manner. These analyses support the notion that ZBM exhibits anti-obesity effects with multiple components acting on multiple targets. Detailed information of the active ingredients and gene symbols are shown in Supplementary Table S6.

### Protein–Protein Interaction Network of ZBM-Related Obesity Targets

**FIGURE 3:**
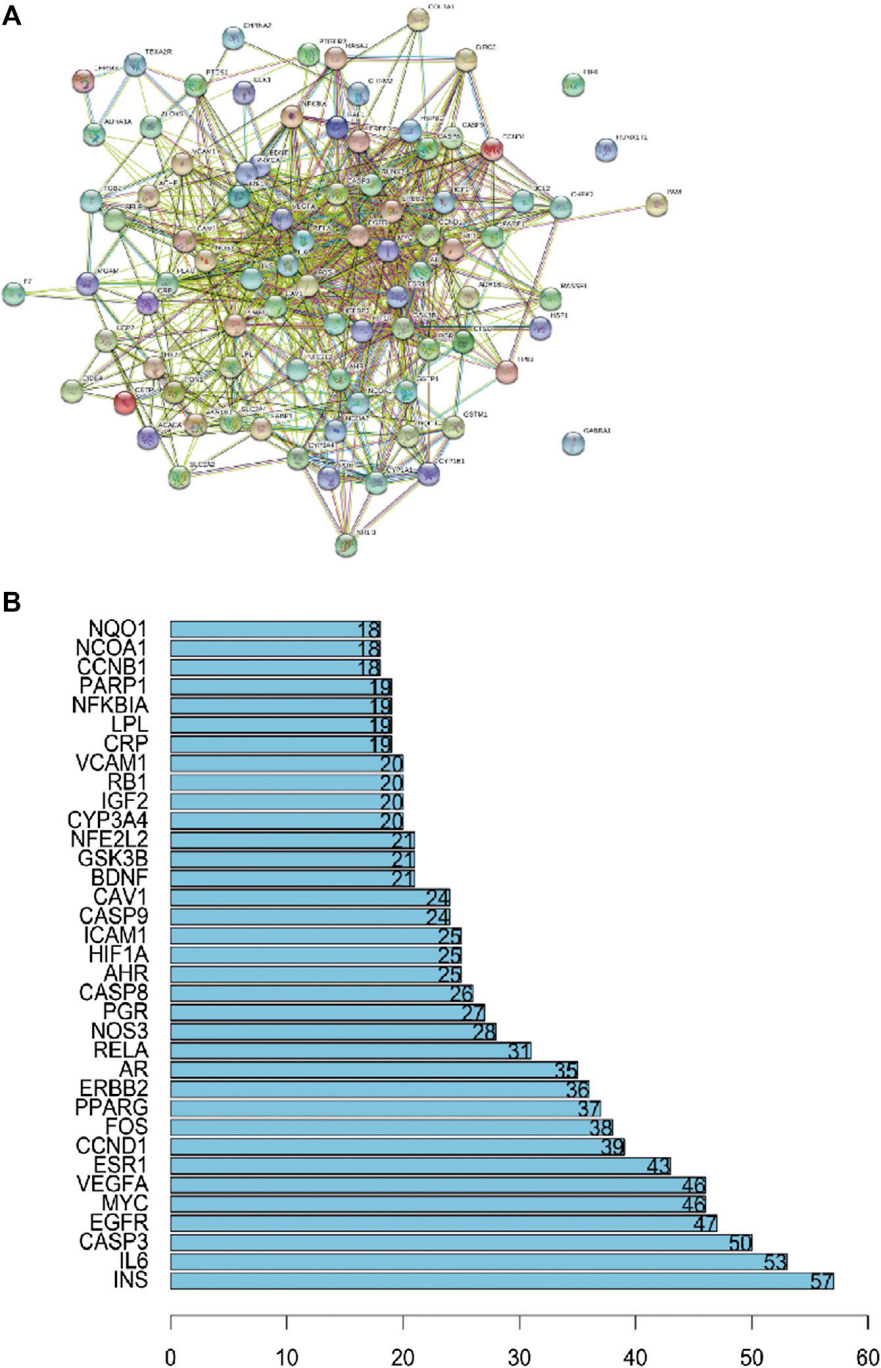
**(A)** Protein–protein interaction network. **(B)** The bar plot of the protein–protein interaction network. The *X*-axis showing the number of neighboring proteins of the target one. The *Y*-axis showing the target protein.

The PPI network was constructed based on the PPIs for the candidate protein targets of ZBM. Figure 3A showed that the PPI network contains 88 nodes and 766 edges. The light blue edges shown in Figure 3A represent the reported interactions curated from databases. Pink edges represent the reported interactions determined by experimental verification. Green edges represent the predicted interactions with the neighborhood genes. Red edges represent the predicted interactions based on gene fusions. Dark blue edges represent the predicted interactions from gene co-occurrence. Yellow edges represent the predicted interactions from the text mining. Black edges represent the predicted interactions from co-expression. Lavender edges represent the predicted interactions based on protein homology. In the PPI network, the nodes with higher degree may be more important in the pharmacological process. Details of the PPI network are shown in Supplementary Table S7. The top 35 proteins in the PPI network may represent the key molecular targets in the anti-obesity effects of ZBM. As shown in Figure 3B, we found that INS, IL-6, and CASP3 are separately linked to other 57, 53, and 50 proteins, respectively. EIF6, RUNX1T1, and GABRA1 have no relationship with other proteins.

### Gene Ontology and Kyoto Encyclopedia of Genes and Genomes Pathway Enrichment Analyses

**FIGURE 4:**
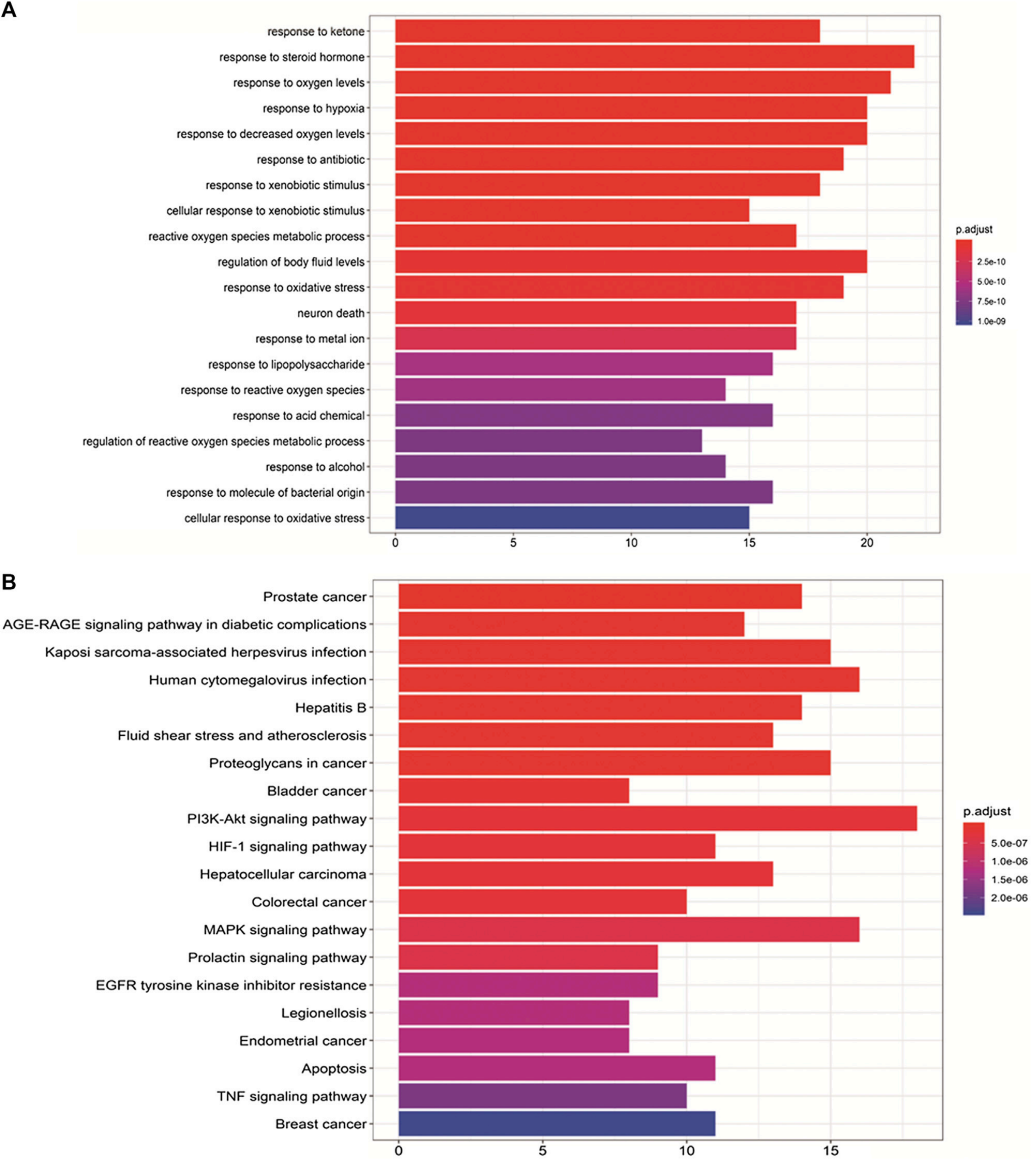
Gene Ontology (GO) and Kyoto Encyclopedia of Genes and Genomes pathway analyses. **(A)** GO analyses of the 88 gene symbols associated with obesity. The *x*-axis showing the significant enrichment in the counts of these terms. The *y*-axis showing the categories of “biological process” in the GO of the target genes (p < 0.01). **(B)** Kyoto Encyclopedia of Genes and Genomes pathway enrichment analyses. The *X*-axis showing the counts of the target symbols in each pathway; the *Y*-axis showing the main pathways (*p* < 0.01).

To verify the biological characteristics of the 88 highlighted targets of ZBM, GO enrichment analysis of putative targets was performed to clarify the relevant biologic processes (*p* < 0.01) as shown in Figure 4A. The *y*-axis represents GO terms, and *x*-axis indicates the number of genes enriched in that term. The color from blue to red indicates the value of p. Adjust (FDR) is getting smaller with greater credibility and importance. Here, we chose the top 20 terms based on their *p*-value. Detailed information of GO analyses is shown in Supplementary Table S8. The results indicated that several biological processes were involved in the anti-obesity effects of ZBM, including the response to ketone (GO: 1901654), response to steroid hormone (GO: 0048545), response to oxygen levels (GO: 0070482), and response to hypoxia (GO: 0001666).

To further identify the potential pathways involved in the anti-obesity effects of ZBM, KEGG pathway enrichment analysis of the 88 genes was performed. Similarly, a total of 20 enriched pathways involved in the anti-obesity effects of ZBM were identified and presented in Figure 4B. KEGG pathway information was presented in detail in Supplementary Table S9. The enriched genes were linked to a variety of signaling pathways, including inflammatory pathways and apoptotic pathways. As shown in Figure 4B, the 25 overlapping gene symbols interacted closely with the pathways involved in apoptosis (hsa04210), PI3K-AKT signaling pathway (hsa04151), and MAPK signaling pathway in diabetic complications (hsa04010), suggesting that these pathways may mediate the anti-obesity effects of ZBM.

### Computational Validation of Selected Ingredient–Target Interactions

**FIGURE 5:**
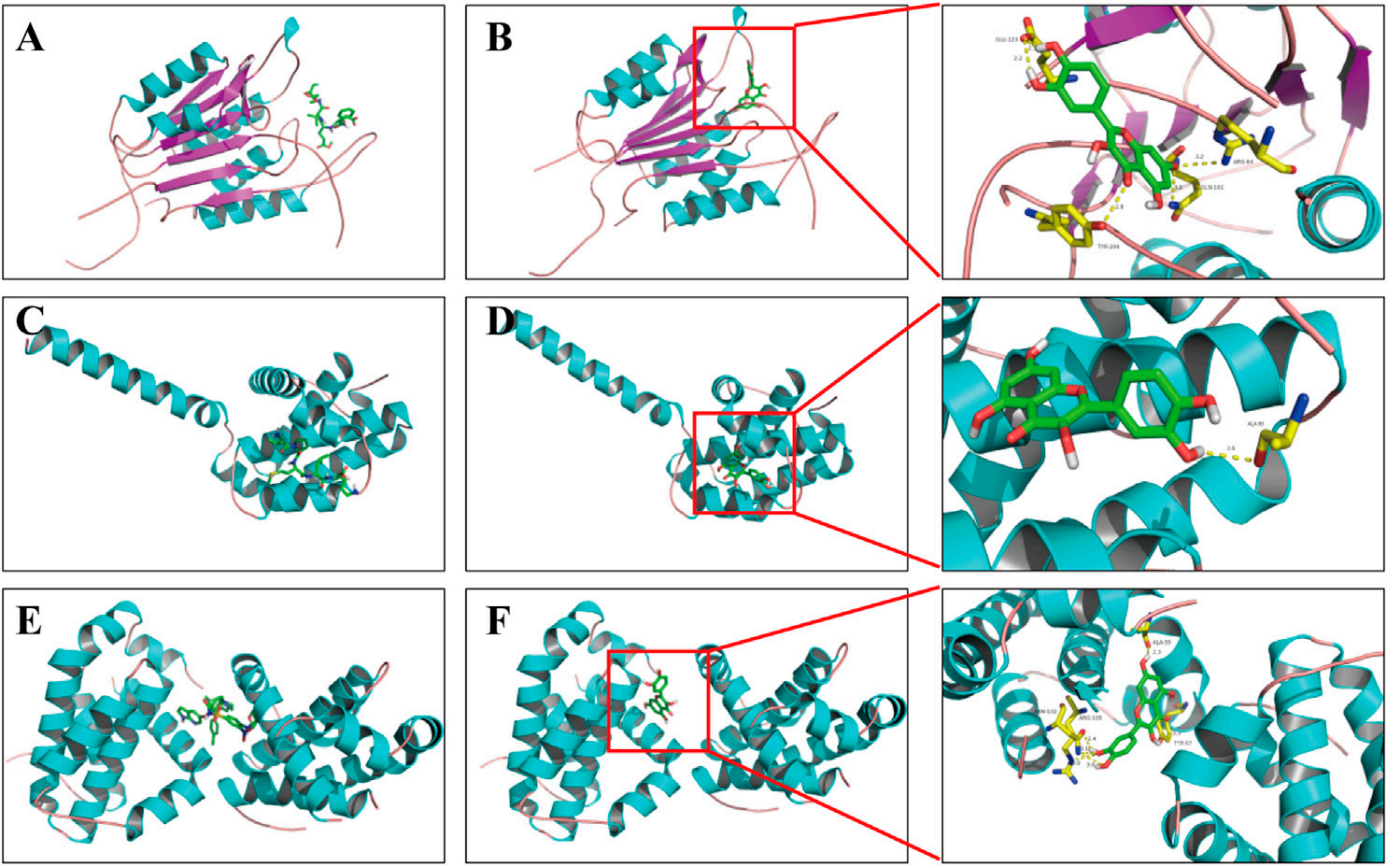
Binding mode of quercetin and apoptosis-related targets. **(A)** Z-DEVD-FMK with caspase 3; **(B)** quercetin with caspase 3; **(C)** ABT-199 with Bcl-2; **(D)** quercetin with Bcl-2; **(E)** peptide V5 with Bax; **(F)** quercetin with Bax. Molecules are represented by a ball-and-stick model, and the hydrogen bonds are represented by a dotted line, and the distance is in angstroms. Atoms C, O, and N are green, red, and blue in color, respectively.

In general, the strength of a ligand bound to a receptor is determined by the number of covalent bonding and the binding affinities (Kuo and Lauffenburger, 1993), and the binding will subsequently affect the protein activity. Here, molecular docking was used to reveal the potential binding mode between the obesity-related apoptotic markers (e.g., caspase 3, Bax, and Bcl-2) and quercetin, a compound with the highest degree of connections in the compound–target network. As shown in Figure 5, quercetin adopted a compact conformation in binding with caspase 3 (Figure 5B), Bax (Figure 5D), and Bcl-2 (Figure 5F), respectively. To verify whether our model is robust and reliable, we performed the docking assay with three inhibitors of caspase 3 (Z-DEVD-FMK, Figure 5A), Bcl-2 (ABT-199, Figure 5C), and Bax (peptide V5, Figure 5E) to each target, respectively. It was found that quercetin and the respective inhibitor of each target bind to the same location to caspase 3, Bcl-2, and Bax, respectively. Moreover, we found that quercetin occupied the hydrophobic pocket composed of the residues GLU123 (bond length: 2.2 Å and 2.1 Å), ARG-64 (bond length: 3.2 Å), GLN-161 (bond length: 3.0 Å), and TYR-204 (bond length: 2.8 Å) in caspase 3 (Figure 5B); the hydrophobic pocket composed of residues ASN-102 (bond length: 3.1, 3.0, and 2.4 Å) , ARG-105 (bond length: 3.0 Å), TYR-67 (bond length: 3.3 Å), and Ala-59 (bond length: 2.3 Å) in Bcl-2 (Figure 5D), as well as the hydrophobic pocket composed of ALA-83 (bond length: 2.8 Å) in Bax (Figure 5F). Quercetin is capable of forming strong hydrophobic bindings with these apoptotic markers. Our analysis suggests that the interactions between these apoptotic markers and quercetin underline the apoptotic effects of ZBM.

### Experimental Validation *in vitro*

**FIGURE 6:**
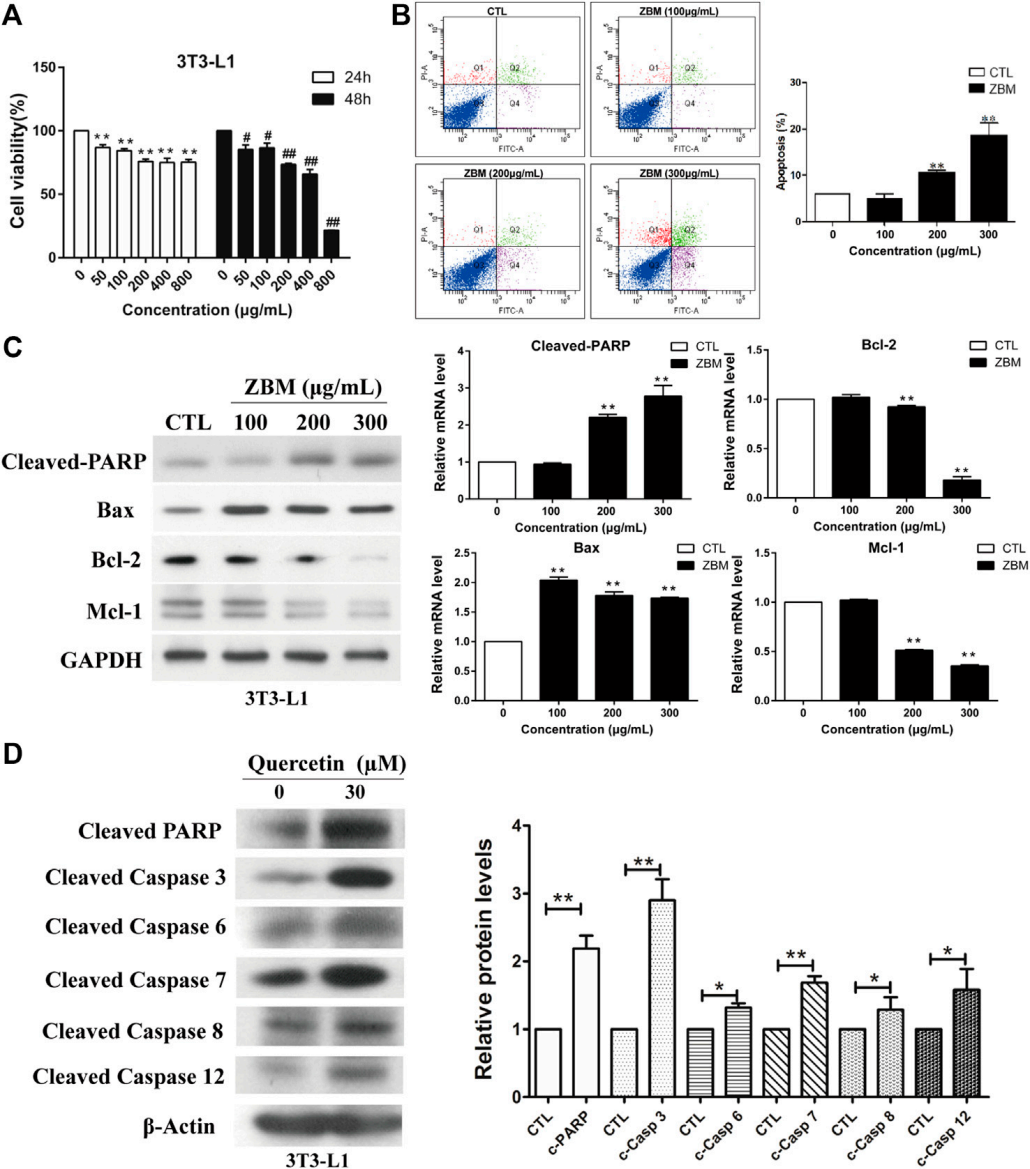
*Zanthoxylum bungeanum* Maxim (ZBM) induces apoptosis in 3T3-L1 adipocytes. **(A)** Cell viability of 3T3-L1 after ZBM treatment at the indicated time points. **(B)** Apoptotic cell distribution and the percentages of apoptotic cells after ZBM treatments. **(C)** Expressions of cleaved poly (ADP-ribose) polymerase (PARP), Bax, Bcl-2, and Mcl-1 in 3T3-L1 cells after ZBM treatment, and the quantitative analysis of the corresponding protein expressions. **(D)** Expressions of cleaved PARP (c-PARP), cleaved caspase 3 (c-casp 3), cleaved caspase 6 (c-casp 6), cleaved caspase 7 (c-casp 7), cleaved caspase 8 (c-casp 8), and cleaved caspase 12 (c-casp 12) in 3T3-L1 cells after quercetin treatment and the quantitative analysis of the corresponding protein expressions. The data are shown as the means ± SEM, n = 3 individual experiments, *p < 0.05, **p < 0.01 compared to control.

The apoptotic effect of ZBM was first validated *in vitro*. We first examined the effect of ZBM on the viability of 3T3-L1 adipocytes, which are the commonly used adipocyte models (Su et al., 2020). Figure 6A showed that ZBM significantly reduced the viability of 3T3-L1 cells after 24 or 48 h treatments. At 200 μg/mL, the cell viability of 3T3-L1 was 75.9% after 24h treatment and was 74.9% after 48h treatment. At 400 μg/mL, the cell viability of 3T3-L1 was 73.5% after 24h treatment and was 65.9% after 48h treatment.

Annexin V-fluorescein isothiocyanate/PI staining was used to assess the percentage of the apoptotic cells after treatments. 3T3-L1 cells were treated by ZBM for 24 h. As shown in Figure 6B, ZBM treatment induced apoptosis in 3T3-L1 cells in a dose-dependent manner. The *in vitro* apoptosis data were further examined in terms of the apoptotic pathways. The mammalian BCL-2 family members Bcl-2 and Mcl-1 are anti-apoptotic proteins (Hardwick et al., 2012), while Bax protein induces apoptosis by increasing cytochrome c release from the mitochondria (Pawlowski and Kraft, 2000). The subsequent collapse of the mitochondrial membrane potential will activate the downstream caspase activities including caspase 6, 7, and 3. Besides, the activation of death receptor activates caspase 8, or ER stress that activates caspase 12, will all lead to the activation of the effector caspase 3 that executes apoptosis in the cells (McIlwain et al., 2015). When cells undergo apoptosis, the activity of poly (ADP-ribose) polymerase (PARP) will be amplified, resulting in high NAD^+^ consumption and depletion of the ATP pools. During apoptosis, caspase will cleave and inactivate PARP (Chaitanya et al., 2010), and hence, the expression of cleaved PARP is commonly used as an indicator for apoptosis. As shown in Figure 6C, ZBM treatment significantly increased protein levels of cleaved PARP and pro-apoptotic proteins Bax, while reducing the levels of anti-apoptotic proteins Bcl-2 and Mcl-1, suggesting that ZBM induces apoptosis in 3T3-L1 cells. Further studies showed that quercetin, a component in ZBM extract, also induced apoptosis in 3T3-L1 cells as indicated by the increased protein levels of cleaved PARP, caspase 3, 6, 7, 8, and 12 as shown in Figure 6D. Our data are in agreement with the system network pharmacology study that highlights the apoptotic function of ZBM in adipocytes.

### Experimental Validation *in vivo*

**FIGURE 7:**
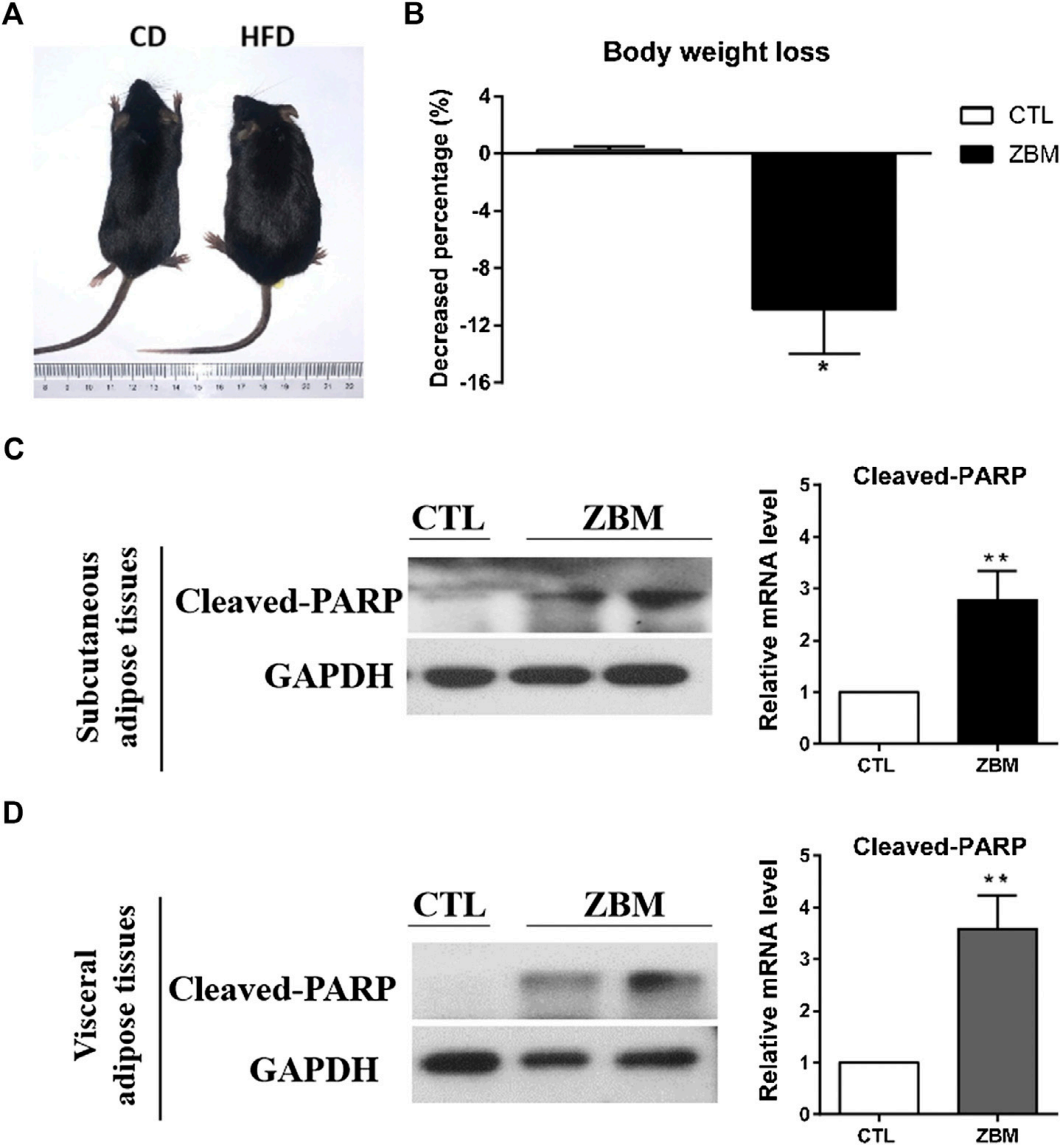
*Zanthoxylum bungeanum* Maxim (ZBM) treatment reduces body weight and induces apoptosis in adipose tissues in the HFD-induced obesity (DIO) mouse model. **(A)** Picture of the CD-fed and the HFD-fed mice. HFD, high-fat diet; CD, comparable control diet. **(B)** Body weight of DIO mice after ZBM treatments. Expression of cleaved PARP in **(C)** subcutaneous adipose tissue and **(D)** visceral adipose tissue in the DIO mice after ZBM treatments. The data are shown as the means ± SEM, n = 6 mice in each group. *p < 0.05 compared to vehicle control group.

We next used the DIO mouse model to examine whether ZBM-induced apoptosis in adipocytes *in vivo* and reduced body weight. We use HFD to induce obesity in the mouse model and a matched CD as control. As shown in Figure 7A, after 8 weeks of dietary intervention, the body weights of mice fed by HFD were significantly higher than those fed by CD, suggesting the DIO mouse model has been established. We then randomly separated the DIO mice into the ZBM treatment group (4 g/kg) or the vehicle control group. After 12 days of treatment, the body weights of the mice in the ZBM group were significantly reduced, while no significant change was observed in the control group (Figure 7B). Furthermore, ZBM treatment also increased the protein levels of cleaved PARP in the subcutaneous adipose tissues (Figure 7C) and in the visceral adipose tissues (Figure 7D) in these mice. Taken together, the *in vivo* study strongly suggests that ZBM induces apoptosis in the adipose tissues and reduces body weight of the DIO mice.

## Discussion

Herbs or herbal compounds always have multiple targets. The efficacy evaluation of an herbal treatment is also challenging because it is composed of many bioactive compounds. Network pharmacology is the ideal tool for the study of the pharmacology of the herbal treatment in disease treatment. The networks can be built using a knowledge-based strategy, computation-based strategy, and experiment-based strategy. Network pharmacology is useful for the discovery of the relationships among the herb, the disease, and the molecular targets on a network basis, and for the understanding of the molecular mechanisms underlying the therapeutic effects of the treatment. In this study, we have first identified the bioactive components in ZBM and the related targets of the ingredients in ZBM and also acquired the known therapeutic targets for obesity and built up the D-I-G-D network. The D-I-G-D network highlights a total of 88 genes that may be the molecular targets of ZBM in the treatment of obesity. Among these 88 genes, many of them are involved in apoptosis such as Bcl-2 and caspase 9, 3, and 8. Indeed, KEGG pathway enrichment analysis of the 88 genes has also highlighted the apoptotic pathway, implying apoptosis is involved in the anti-obesity effect of ZBM. We have also established the PPI network that highlights a total of 35 proteins that may represent the critical molecular targets that mediate the anti-obesity effects of ZBM. Among these molecular targets, caspase 3 is linked to other 50 proteins, again suggesting the importance of caspase 3 in mediating the anti-obesity effect of ZBM. We have also used both *in vitro* and *in vivo* studies to validate whether ZBM induces apoptosis in adipocyte and reduces obesity. Indeed, our data strongly suggest that ZBM induces apoptosis in subcutaneous adipose tissues and visceral adipose tissues and reduces body weight of the DIO mouse model. Mechanistic studies with 3T3-L1 cell model suggest that ZBM activates the apoptotic pathways. The apoptotic effect of ZBM is further suggested by quercetin, one of its bioactive components that induces both intrinsic and extrinsic apoptotic pathways in the 3T3-L1 cell model.

Apoptosis is a normal phenomenon of cell death for maintaining homeostasis, which can reduce the number of adipocytes. Induction of apoptosis has been considered as a feasible way to reduce the number of adipocytes in obese patients (Zhang and Huang, 2012). Experimental studies show that some natural compounds reduce obesity by inducing adipocytes apoptosis. For example, genistein reduces body weight *via* induction of apoptosis of adipose tissues in the mouse models (Kim et al., 2006; Rayalam et al., 2008). In our study, we suggest that quercetin is one of the bioactive compounds in ZBM that induces adipocyte apoptosis. Further study is needed to find out other bioactive compounds in ZBM that mediate the apoptotic effects and examine whether synergy exists among these compounds. However, some studies suggest that targeted induction of adipocyte apoptosis may increase blood lipid levels and ectopic lipid storage (Colitti and Grasso, 2014). Nevertheless, the release of fatty acid from adipocytes can also activate peroxisome proliferator-activated receptor delta to induce fatty acid oxidative genes and increase fatty acid oxidation and energy expenditure (Kim et al., 2016). In our study, ZBM treatment significantly reduces body weight of the DIO mice. In the future, other parameters of the ZBM-treated mouse model can be examined including the serum fatty acid levels; lipid contents in major organs such as liver, kidney and heart; and the circulating inflammatory marker levels. Furthermore, the energy expenditure of the mice after ZBM treatment can also be studied. A well-defined dosage of ZBM that induces adipocyte apoptosis and reduces body weight without causing ectopic lipid storages can be developed as health product or therapeutics to reduce obesity and control body weight.

## Conclusion

ZBM is used as medicine and is also used as spice for cooking, it is safe for human consumption. With advancement in technology, we have revealed the apoptotic effect of ZBM in adipocytes with network pharmacology, which is also validated by *in vitro* studies. Moreover, our *in vivo* study with the DIO mouse model further suggests the pro-apoptotic effect in adipocytes and the anti-obesity effect of ZBM. Development of ZBM as food supplement or therapeutics to reduce obesity and control body weight will benefit the overweight and obese populations.

## Data Availability Statement

All datasets presented in this study are included in the article/Supplementary Material.

## Ethics Statement

The animal study was reviewed and approved by the Research Ethics Committee of the Hong Kong Baptist University.

## Author Contributions

TS, YW, and SY performed the majority experiments. KZ, TJ, and MZ participated in several experiments. TS and HK interpreted the data, drafted, and finalized the manuscript. All authors have read and approved the final manuscript.

## Conflict of Interest

The authors declare that the research was conducted in the absence of any commercial or financial relationships that could be construed as a potential conflict of interest.
