# Supplementary figures and images for: Network Pharmacology-Based Strategy for the Investigation of the Anti-Obesity Effects of an Ethanolic Extract of Zanthoxylum bungeanum Maxim
Source: Front Pharmacol. 2020 Nov 13;11:572387. doi: 10.3389/fphar.2020.572387 (PMC7751641; doi:10.3389/fphar.2020.572387)

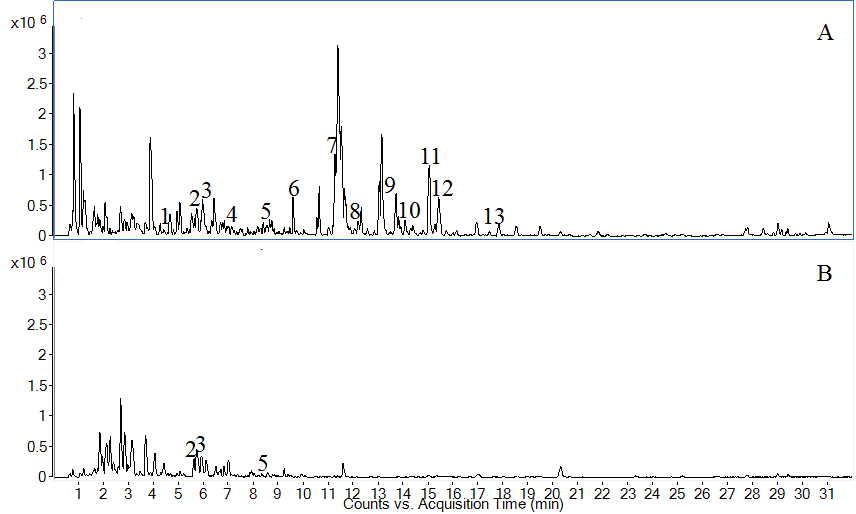

Supplement: Supplementary file 1 [file Image1_v1.TIF]

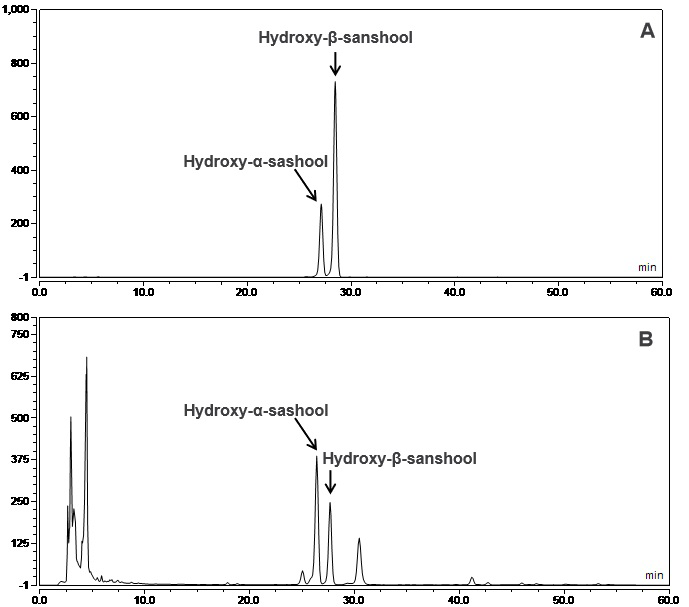

Supplement: Supplementary file 2 [file Image2_v1.TIF]
