# Supplementary material for: Network Pharmacology-Based Strategy for the Investigation of the Anti-Obesity Effects of an Ethanolic Extract of Zanthoxylum bungeanum Maxim
Source: Front Pharmacol. 2020 Nov 13;11:572387. doi: 10.3389/fphar.2020.572387 (PMC7751641; doi:10.3389/fphar.2020.572387)
Supplement: Supplementary file 3 [file Table1_v1.PDF]

Supplementary Table S1 | Compounds identified from ZBM mature pericarp extracts.

Peak No.	Identity	Formula	T_R (min)	$[M+H]^+$ Mass accuracy (ppm)	$[M-H]^-$ Mass accuracy (ppm)	Mean measured mass(Da)	Theoretical exact mass (Da)	Quasimolecular ion
1	Herniarin	$C_{10}H_8O_3$	4.391	-1.27	-	177.0559	177.0546	$[M+H]^+$
2	Rutin	$C_{27}H_{30}O_{16}$	5.727	-2.67	-1.62	611.1668/ 609.1494	611.1607/ 609.1461	$[M+H]^+$/ $[M-H]^-$
3	Hyperoside	$C_{21}H_{20}O_{12}$	5.91	-1.86	-	465.1062/ 463.0902	465.1028/ 463.0882	$[M+H]^+$/ $[M-H]^-$
4	Tamarixetin	$C_{16}H_{12}O_7$	7.22	-3.21	-	317.0688	317.0656	$[M+H]^+$
5	Quercetin	$C_{15}H_{10}O_7$	5.725	-2.94	-0.74	303.0528/ 301.0361	303.0499/ 301.0354	$[M+H]^+$/ $[M-H]^-$
6	Skimmianine	$C_{14}H_{13}NO_4$	9.589	-3.56	-	260.0954	260.0917	$[M+H]^+$
7	Hydroxy-alpha-Sanshool	$C_{16}H_{25}NO_2$	11.252	-3.55	-	286.1813	286.1783	$[M+Na]^+$
8	N-benzoyltyramine	$C_{15}H_{15}NO_2$	12.304	-3.75	-	242.1214	242.1176	$[M+H]^+$
9	Simulansine	$C_{20}H_{25}NO_3$	13.695	-1.23	-	328.1917	328.1907	$[M+H]^+$
10	Tambulin	$C_{18}H_{16}O_7$	14.149	-3.67	-	345.1006	345.0969	$[M+H]^+$
11	Sanshool	$C_{16}H_{25}NO$	15.036	-3.58	-	248.2037	248.2009	$[M+H]^+$
12	Zanthosimuline	$C_{20}H_{23}NO_2$	15.413	-0.21	-	310.1459	310.1802	$[M+H]^+$
13	gamma-sanshool	$C_{18}H_{27}NO$	17.825	-2.57	-	274.2192	274.2165	$[M+H]^+$
