# Supplementary material for: Network Pharmacology-Based Strategy for the Investigation of the Anti-Obesity Effects of an Ethanolic Extract of Zanthoxylum bungeanum Maxim
Source: Front Pharmacol. 2020 Nov 13;11:572387. doi: 10.3389/fphar.2020.572387 (PMC7751641; doi:10.3389/fphar.2020.572387)
Supplement: Supplementary file 12 [file Table10_v1.DOC]

**Table 1 | A total of twenty active ingredients were obtained from ZBM**

| **No.** | **CAS** | **Name** | **References** |
| --- | --- | --- | --- |
| 1 | 7149-26-0 | Linalyl anthranilate | (Dharmawan et al., 2008) |
| 2 | 469-92-1 | CLOVENE | (Cardeal et al., 2006) |
| 3 | 577-27-5 | Ledol | (Dhami et al., 2019) |
| 4 | 73464-47-8 | Beta-Gurjunene | (Cardeal et al., 2006) |
| 5 | 13744-15-5 | Beta-Cubebene | (Sekiwa-Iijima et al., 2002) |
| 6 | 83-95-4 | Skimmianin | (Abe et al., 1973) |
| 7 | 520-34-3 | Diosmetin | (Cardeal et al., 2006) |
| 8 | 6750-60-3 | Spathulenol | (Shafi et al., 2000) |
| 9 | 83-46-5 | Beta-sitosterol | (Tsai et al., 2000) |
| 10 | 482-36-0 | Hyperoside | (Ahsan et al., 2000) |
| 11 | 531-59-9 | Herniarin | (Sekiwa-Iijima et al., 2002) |
| 12 | 581-31-7 | Suberosin | (Ahsan et al., 2000) |
| 13 | 17156-84-2 | Oleic acid | (Li et al., 2001) |
| 14 | 117-39-5 | Quercetin | (Ahsan et al., 2000) |
| 15 | 153-18-4 | Rutin | (Cho et al., 2003) |
| 16 | 83883-10-7 | Hydroxy-α-sashool | (Rong et al., 2016; Li et al., 2019) |
| 17 | 97465-69-5 | Hydroxy-β-sanshool | (Rong et al., 2016; Li et al., 2019) |
| 18 | 78886-66-5 | Hydroxy-γ-sanshool | (Rong et al., 2016; Li et al., 2019) |
| 19 | 138-86-3 | limonene | (Sun et al., 2020) |
| 20 | 78-70-6 | linalool | (Sun et al., 2020) |
